# Novel Transcriptomic Signatures in Fibrostenotic Crohn’s Disease: Dysregulated Pathways, Promising Biomarkers, and Putative Therapeutic Targets

**DOI:** 10.1093/ibd/izaf021

**Published:** 2025-02-20

**Authors:** Animesh Acharjee, Uday Shivaji, Giovanni Santacroce, Sarah Akiror, Louisa Jeffery, Csilla Varnai, Gary Reynolds, Davide Zardo, Snehali Majumder, Asma Amamou, Georgios V Gkoutos, Marietta Iacucci, Subrata Ghosh

**Affiliations:** Institute of Cancer and Genomic Sciences, University of Birmingham, Birmingham, UK; Centre for Health Data Research, University of Birmingham, Birmingham, UK; Gastroenterology, Queen Elizabeth Hospital, University Hospitals Birmingham NHS Foundation Trust, Birmingham, UK; Institute of Immunology and Immunotherapy, University of Birmingham, Birmingham, UK; APC Microbiome Ireland, College of Medicine and Health, University College Cork, Cork, Ireland; Centre for Liver and Gastrointestinal Research, University of Birmingham, Birmingham, UK; Institute of Immunology and Immunotherapy, University of Birmingham, Birmingham, UK; Institute of Cancer and Genomic Sciences, University of Birmingham, Birmingham, UK; Centre for Liver and Gastrointestinal Research, University of Birmingham, Birmingham, UK; Department of Pathology, San Bortolo Hospital, Vicenza, Italy; Institute of Immunology and Immunotherapy, University of Birmingham, Birmingham, UK; APC Microbiome Ireland, College of Medicine and Health, University College Cork, Cork, Ireland; APC Microbiome Ireland, College of Medicine and Health, University College Cork, Cork, Ireland; Institute of Cancer and Genomic Sciences, University of Birmingham, Birmingham, UK; Centre for Health Data Research, University of Birmingham, Birmingham, UK; Gastroenterology, Queen Elizabeth Hospital, University Hospitals Birmingham NHS Foundation Trust, Birmingham, UK; Institute of Immunology and Immunotherapy, University of Birmingham, Birmingham, UK; APC Microbiome Ireland, College of Medicine and Health, University College Cork, Cork, Ireland; APC Microbiome Ireland, College of Medicine and Health, University College Cork, Cork, Ireland

**Keywords:** fibroblast, immune cell, intestinal fibrosis, machine learning, single-cell transcriptomic

## Abstract

**Background:**

Fibrosis is a common complication in Crohn’s disease (CD), often leading to intestinal strictures. This study aims to explore the transcriptomic signature of fibrostenotic ileal CD for a comprehensive characterization of biological and cellular mechanisms underlying intestinal fibrosis.

**Methods:**

Nine CD patients undergoing surgery for fibrotic ileal strictures were prospectively recruited. RNA was extracted from fresh resected samples for bulk transcriptomics. Differentially expressed genes (DEGs) were identified (adj. *P* value < .05), and machine learning analyses were employed to compare gene expression patterns between strictures and non-strictured margins. Pathway enrichment analysis pinpointed relevant pathways. Furthermore, a random forest model was constructed to evaluate the significance of targeted genes. Relevant genes were subsequently validated through qPCR and further analyzed using a publicly available bulk RNA-seq dataset (GSE192786). Single-cell RNA sequencing (scRNA-seq) analysis was performed using the 10× Chromium Controller platform.

**Results:**

Bulk transcriptomics revealed unique transcriptomes with 81 DEGs, 64 significantly up-regulated, and 17 down-regulated in strictures compared to non-strictured margins. Up-regulated genes were mainly associated with inflammation, matrix and tissue remodeling, adipogenesis and cellular stress, while down-regulated genes were linked to epithelial barrier integrity. *LY96, AKAP11, SRM, GREM1*, *EHD2*, *SERPINE1*, *HDAC1*, and *FGF2* showed high specificity for strictures. scRNA-seq linked up-regulated *GREM1* exclusively to fibroblasts, while *EHD2* and *FGF2* showed upregulation in both fibroblasts and endothelial cells. *LY96* and *SRM* were expressed by immune cells, whereas *HDAC1, AKAP11, and SERPINE1* showed low expression across all cellular subsets.

**Conclusions:**

This study comprehensively characterizes resected CD ileal strictures, elucidating main dysregulated pathways and identifying promising biomarkers and putative therapeutic targets.

## Graphical Abstract

**Graphical Abstract:**
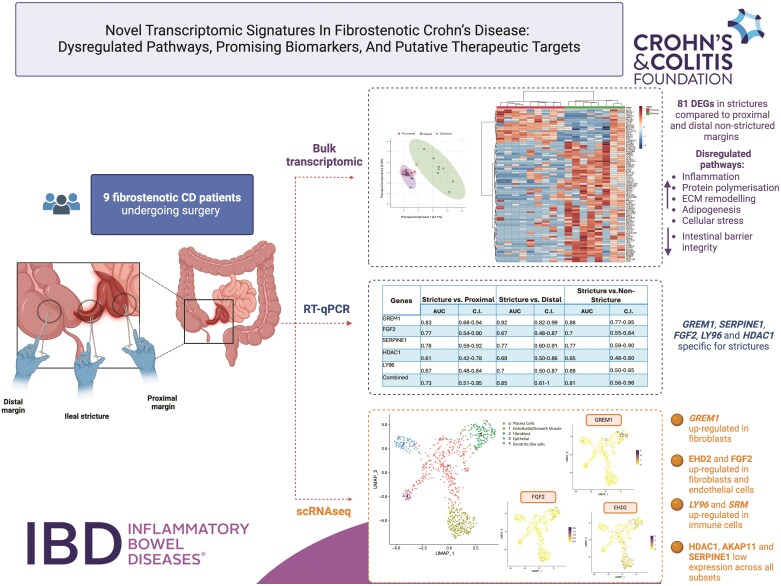

**Key messages**

**What Is Already Known?**

Intestinal fibrosis is a severe complication of Crohn’s disease, often leading to strictures that require surgery. While current understanding identified key players in the fibrotic process, no biomarkers or anti-fibrotic therapies have reached clinical practice.

**What Is New Here?**

This study identifies eight key genes (*LY96, AKAP11, SRM, GREM1, EHD2, SERPINE1, HDAC1*, and *FGF2*) specific for strictures, with single-cell RNA sequencing revealing cell-specific gene expression patterns.

**How Can This Study Help Patient Care?**

The identified genes and pathways can serve as biomarkers for early detection of strictures and guide the development of targeted therapies, potentially improving outcomes for patients with Crohn’s disease.

## Introduction

Intestinal fibrosis is a common complication of Crohn’s disease (CD),^1^ a disorder characterized by chronic inflammation. Chronic inflammation prompts the inappropriate deposition of extracellular matrix (ECM), leading to strictures observed in over 50% of CD patients.^2^ The fibrostenotic CD phenotype (Montreal B2) is associated with adverse outcomes, including high morbidity, hospitalization, surgery and poor quality of life for patients, and high costs of care for health system.^3^

Significant efforts have been devoted to unraveling and addressing the puzzle of intestinal fibrosis. An orchestra of players has been identified to be involved in this process. Among these, myofibroblasts stand out as the main cellular actors, leading the production and accumulation of ECM under the influence of several cytokines, especially the transforming growth factor (TGF)-β. Nevertheless, the complex fibrotic network encompasses several other cell types, such as epithelial cells, endothelial cells, and innate and adaptive immune cells, all interconnected with stromal cells to drive fibrotic stenosis.^4^ Our recent histological analysis has revealed the prominent role of smooth muscle hyperplasia of submucosa and hypertrophy of muscularis propria, along with chronic inflammation, in small bowel fibrotic strictures.^5,6^ Moreover, a pro-fibrotic role for mesenteric adipose tissue has been proposed. A phenomenon called “creeping fat,” defined as the expansion of adipose tissue around the inflamed and fibrotic intestine, has been related to microbes-driven tissue remodeling via M2 macrophages.^7^ The immune cells and adipocytes in creeping fat can produce many pro-fibrotic cytokines, adipokines, growth factors, and fatty acids, promoting the fibrotic process.

Despite the enhanced comprehension of the mechanisms underlying intestinal fibrosis and strictures, no biomarkers or anti-fibrotic therapy has made its way into clinical practice for stricturing CD.^8^ OMIC techniques hold promise in advancing our understanding of intestinal fibrosis in CD, offering a holistic perspective of this process and paving the way for the discovery of novel therapeutic targets and the development of effective therapeutic agents.^9^

Recently, Mukherjee et al. conducted a single-cell RNA sequencing analysis of fibroblasts in human stricturing CD, revealing fibroblast heterogeneity in the mucosa and submucosa.^10^ Their study highlighted the central signaling role of CXCL14+ and MMP/WNT5A+ fibroblasts. In addition, the adhesion molecule CDH11 was found to be broadly up-regulated, representing a potential therapeutic target. Another recent study by Zhang et al. identified a distinct population of fibroblasts (FAP+ and TWIST1+) in human fibrotic sites.^11^ These fibroblasts were identified as major producers of ECM and showed significant interaction with CXCL9+ macrophages. Pharmacological inhibition of TWIST1 demonstrated potential in mitigating fibrosis in mouse models. While these pioneering studies have significantly advanced our understanding of stricturing CD, they are predominantly focused on fibroblasts heterogeneity within stricturing samples and do not take into account the different stages of intestinal strictures. The progression stages of inflammation and fibrosis at varying stages can profoundly influence the genetic environment and cellular behavior.

Building on these foundations, our prospective study aimed to delve into the transcriptomic signatures of fibrostenotic distal ileal Crohn’s disease, with a specific focus on end-stage fibrosis devoid of active inflammation, confirmed through macroscopic and microscopic examination. We sought to discern the differential gene expression between strictured and adjacent non-strictured bowel segments and identify pathways and cellular components that hold promise as biomarkers and therapeutic targets for intestinal fibrosis.

## Methods

### Ethical Approval

The study was registered and approved by the Heath Research Authority, UK, and hospital research governance (ref. number RRK6508).

### Study Population

Nine patients with fibrostenotic CD involving the distal small bowel and undergoing surgery due to stricture-related obstruction were prospectively enrolled at University Hospitals Birmingham, UK between June 2018 and January 2020. The fibrostenotic phenotype was carefully assessed and confirmed by a multidisciplinary assessment based on clinical details, blood biomarkers—C-reactive protein and fecal calprotectin—and imaging (ie, colonoscopy and cross-sectional imaging by magnetic resonance enterography). All relevant demographic and clinical data were collected and managed using REDCap electronic data capture tools hosted at the University of Birmingham, UK.

### Samples Collection

Resected specimens were harvested directly from the operating theater and immediately delivered to pathologists, who meticulously dissected fresh samples from 3 sites: 1 from the ileal stricture, 1 from the non-strictured ileal proximal margin, and 1 from the non-strictured distal margin, usually involving the ileocecal valve. Pathologists macroscopically identified the segments of the strictured intestine. Study samples were freshly sent to the laboratory for further immediate processing. Pathologists confirmed the absence of inflammation in resected specimens using the Robarts Histopathology Index (RHI) and only patients with an RHI score ≤ 3 (without lamina propria or epithelial neutrophils) were included in the analysis.

### RNA Bulk Transcriptomic Sequencing and Analysis

Tissue samples from different segments were stored in RNA later for 24 h at 4 °C before RNA extraction and purification using the RNeasy on-column extraction kit (Qiagen). RNA was quantified by Qubit fluorometer (Life Technologies), and 0.5 µg of RNA was used to generate uniquely indexed cDNA libraries with the QIAseq UPX 3′ transcriptome kit according to manufacturer’s instructions. Libraries were quantified and quality-controlled using the QIAseq Library Quant Assay Kit and tapestation analysis before being sequenced on the Miseq and Nextseq Illumina platforms, with a sequencing depth of 1–3 million reads/sample. FastQ files were obtained through BaseSpace and processed using CLC Genomics Workbench (Qiagen) for de-multiplexing, alignment, quantification, and normalization of reads.

Bioinformatics analysis of the RNA sequencing data involved DEG analysis for each pairwise comparison—ileal stricture versus non-strictured proximal margin, ileal stricture versus non-strictured distal margin and non-strictured proximal margin vs non-strictured distal margin. Genes with an adjusted *P* value < .05 and a fold change of 2 were considered differentially expressed. Normalized transcripts per million were log-transformed with a pseudocount of 1, and transcripts with < 1 transformed count in any sample were excluded, as transcripts with low variance. DEG analysis was conducted using the limma package with a false discovery rate corrected *P* value < .05 considered significant.

#### Partial Least Squares Discriminant Analysis (PLS-DA)

Partial least squares discriminant analysis (PLS-DA) modeling was performed, and a Variable Importance in Projection (VIP) score > 1 was used for gene selection. Model statistics, including the sum of squares for the selected component (R2) and the predictive ability (Q2), were quantified to assess model performance and avoid overfitting through cross-validation.

#### Random forest

Highly connected genes were combined and their ability to associate with stricture versus nonstricture was assessed using the Random Forest Method of Area under the curve (AUC) analysis. In brief, Random Forest is a popular machine-learning algorithm used for tasks like classification and regression.^12,13^ It works by creating multiple decision trees during training and combining their outputs for the final prediction. For classification problems, it selects the most frequently predicted class (eg, stricture vs non stricture), while for regression tasks, it averages the predictions from all the trees. One of its key strengths is its ensemble approach, which merges predictions from multiple trees to enhance accuracy and minimize overfitting. This capability is further enhanced through a process called Bagging (Bootstrap Aggregation), where each tree is trained on a random subset of the dataset. This technique helps reduce variability and improves the stability of the model.

#### Sample size estimation

In addition, a validation experiment was designed to estimate the required number of samples to capture the effect size of selected genes.

Synthetic data were generated using a multivariate log-normal distribution to create datasets. Subsets with specific sample sizes (eg, 5, 10, 15, 50, 100, etc.) were selected for analysis. In the case of stricture versus non stricture two-group classification, separate datasets were generated for each group at the specified sample sizes. A defined Cohen’s d effect size was applied to one group and its highly correlated variables. A one-way ANOVA was performed on each variable to compare intra-group and inter-group variances, producing *P* values to identify variables with statistically significant differences in variance. Further details can be found in Acharjee et al.^13^

#### Gene set enrichment analysis

To elucidate the biological significance and pathways, enrichment analysis was performed using EnrichR, leveraging Gene ontology biological pathways, Wiki pathways, and Kyoto Encyclopaedia of Genes and Genomes (KEGG).

### Quantitative Polymerase Chain Reaction Validation

Quantitative Polymerase Chain Reaction was used to validate selected genes from bulk RNA-seq. The subset of genes analyzed through qPCR was based on the effect size of the genes and literature evidence. Samples from nine patients considered in bulk RNA-seq and additional samples from 5 other patients (a total of 14 patients) were used. Total RNA was extracted from intestinal samples using the RNeasy on-column extraction kit (Qiagen), and cDNA was obtained through reverse transcription of RNA using an iScript kit (BioRad, Hercules, CA, USA). mRNA expressions were detected with Taqman Real-time PCR assay. β-Actin was used as housekeeping gene. Primers were obtained from PrimerBank. Relative mRNA expression was calculated using the 2-ΔΔCt method. Statistical analysis was performed using the Kruskal–Wallis non-parametric test, and data was presented as a median with a 95% confidence interval.

### Analysis of Publicly Available RNA-seq Data

For further validation, the differential expression of genes identified by our analysis was examined in a public bulk RNA-seq dataset of 40 CD biopsies, that is, 19 fibrotic and 21 non fibrotic (GSE192786), obtained from the Gene Expression Omnibus (GEO, https://www.ncbi.nlm.nih.gov/geo/). Random Forest Method of AUC analysis was applied to evaluate the ability of these genes in distinguishing stricture versus non stricture.

### scRNA-seq and Analysis

In a cohort of 3 fibrostenotic CD patients, scRNA-seq was performed to characterize ileal stricture versus non-strictured proximal margin. The strictured segments were harvested from the operating theater and transported for processing without delay.

Tissue samples were stored overnight at 4 °C in MACS tissue storage solution (Miltenyi Biotec) containing penicillin-streptomycin, ampicillin, and gentamicin 1%. The tissue mass was recorded and washed using cold PBS. Subsequently, the tissue was sectioned into small pieces of approximately 1 × 1 mm^2^ or less and transferred into gentleMACS C tubes containing digestion media (2.5% FCS, RPMI 1640, liberase, deoxyribonuclease I, penicillin-streptomycin, ampicillin and gentamicin). Digestion was performed at 37 °C shaking at approximately 0.1 g for 20–30 min, followed by additional dissociation using the miltenyi gentleMACS dissociator. Digested tissue was then washed with 10% FBS and RPMI, and filtered through a 100 µm cell strainer. The obtained single-cell suspension was centrifuged to remove debris. Isolated cells were counted, stained with propidium iodide to detect dead cells, and sorted using the FACSAria machine (BD Biosciences). Live cells were collected in EDTA and Mg^2+^ free PBS and processed for cDNA synthesis and library preparation using Chromium Single Cell 3′ Library & Gel Bead Kits (10× genomics) per the manufacturer’s instructions. The cDNA was quantified and quality-controlled before being sequenced using Illumina NextSeq500/550High-Outputv2.5Kit to obtain 5000 cells per sample.

Pooled single-cell cDNA library samples were sequenced using adapter sequences as described in Table S1. Sequencing libraries from Illumina 10× Chromium were processed using CellRanger v3.1.0 (10× Genomics). Samples were demultiplexed and converted to fastq format using CellRanger’s mkfastq command. Sequencing reads were mapped onto the human reference genome—GRCh38 (v3.0.0) —and raw read counts in genes were computed using CellRanger’s count command.

The analysis of the count data was then performed using Seurat v3.2.2. Cells with 200–2500 RNA features and a <5% mitochondrial RNA content was retained for further analysis. Data from different samples were merged using the Standard Workflow for data integration by Seurat. UMAP clustering and Principal Component Analysis (PCA) were used to cluster cells. Cell types were identified from cluster markers using the FindAllMarkers() function. Clusters closely apposed in the UMAP_1 VS UMAP2 plot and containing cells with markers resembling fibroblasts, epithelial cells, endothelial cells, and dendritic cells were further subclustered. Scatter plots of gene expression levels across cells were generated using the FeaturePlot() function (Seurat).

## Results

### Population Characteristics

Nine CD patients were included in the study, with a median age of 37 [range19-63], F:M ratio 3:6. According to the Montreal Classification, all patients were classified as having a fibrotic B2 behavior and L1 phenotype, confirmed at a multidisciplinary meeting of IBD gastroenterologists, colorectal surgeons, and histopathologist. Table 1 provides information on the main characteristics of the study population at enrollment.

**Table 1.** Population characteristics.

Fibrostenotic CD patients undergoing surgery, *N* = 9	
Clinical characteristics	
Age (years), median (IQR)	37 (25-55)
F/M	3/6
Disease duration (years) median (IQR)	14 (3.5-28.5)
Montreal classification	
*Age at diagnosis, *n* (%)*	
A1 (<16 years)	2 (22)
A2 (17-40 years)	7 (78)
A3 (>40 years)	0
*Location, *n* (%)*	
L1 (ileal)	5 (56)
L2 (colonic)	0
L3 (ileocolonic)	4 (44)
*Behavior, *n* (%)*	
B1 (non-stricturing, non-penetrating)	0
B2 (stricturing)	9 (100)
B3 (penetrating)	0
Perianal disease	1 (11)
Previous bowel surgery for CD, *n* (%)	2 (22)
Medication before surgery	
Mesalamine, *n* (%)	1 (11)
Steroid, *n* (%)	3 (33)
Immunosuppressive, *n* (%)	3 (33)
Anti-TNF, *n* (%)	3 (33)
Vedolizumab, *n* (%)	0
Ustekinumab, *n* (%)	2 (22)
Bio-experienced, *n* (%)	3 (33)
Laboratory results before surgery	
Hb g/L, median (IQR)	134 [127.4-139]
CRP mcg/mL, median (IQR)	16 [2.5-30]
Albumin g/dL, median (IQR)	43 [38.5-44]
Fecal calprotectin, median (IQR)	128 (82-248)
Type of surgery	
Ileo-caecal resection, *n* (%)	3 (33)
Right hemicolectomy, *n* (%)	2 (22)
Ileal resection, *n* (%)	4 (44)

Abbreviations: CD, Crohn’s disease; CRP, C-reactive protein; F, female; Hb, hemoglobin; IQR, interquartile range; M, male; TNF, tumor necrosis factor.

### Bulk Transcriptomic Profile of Fibrotic Strictures

#### Gene expression profiles between ileal stricture and non-strictured margins

**Figure 1.**
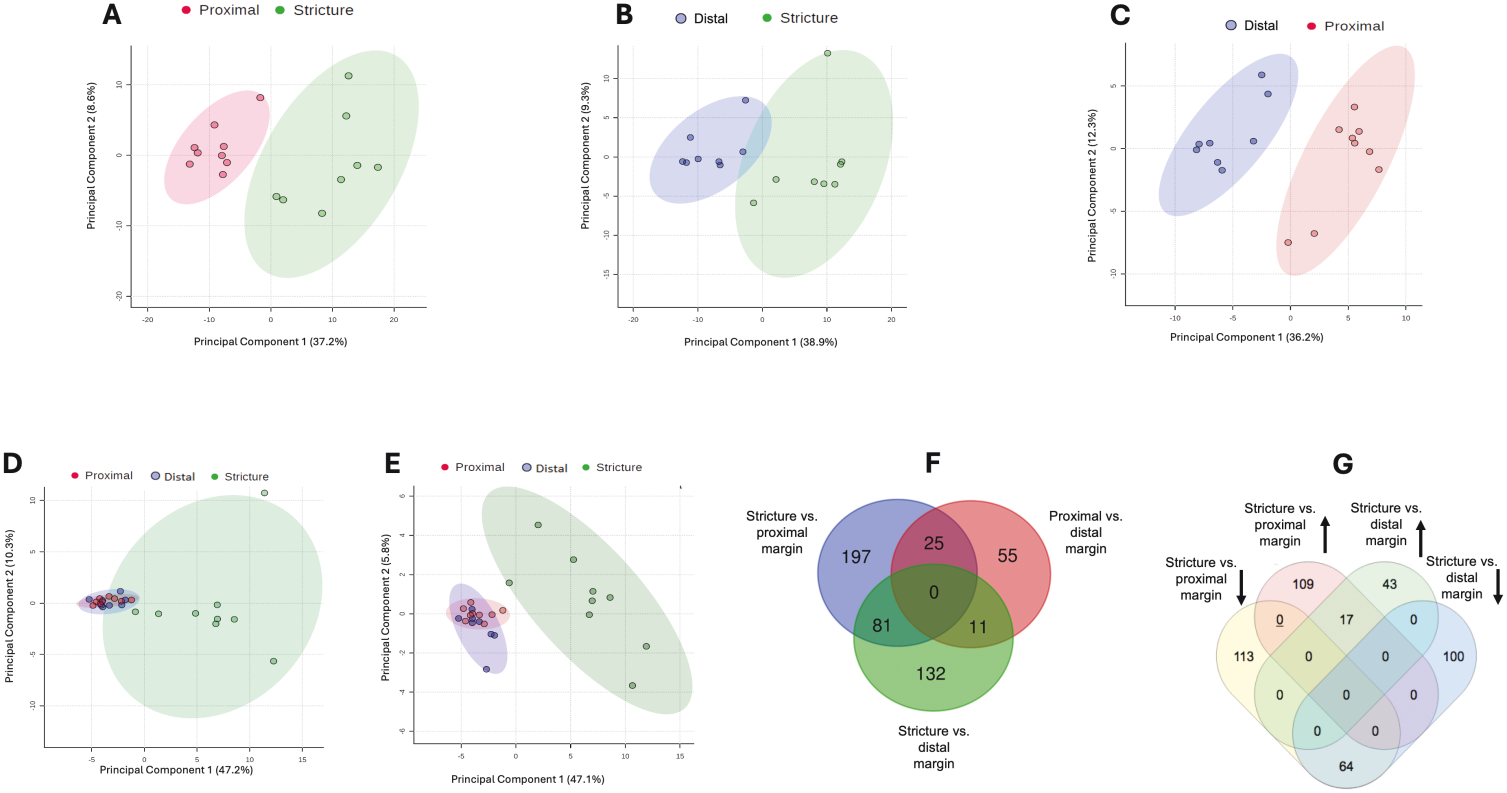
Differentially expressed genes (DEGs) analysis between stricture, non-strictured proximal margin, and non-strictured distal margin. (A-C) Principal component analysis (PCA) score plots performed on the differentially expressed transcriptome datasets demonstrating clustering and variation of subjects within cohorts, that is, stricture versus proximal margin (A), stricture versus distal margin (B), and proximal versus distal margin (C). The dots represent samples and are colored according to the subject cohort. Ellipses represent 95% confidence. Results are plotted according to the PC1 and PC2 scores, with the percent variation explained by the respective axis. D, Principal component analysis score plot for combined analysis of stricture, proximal and distal samples. E, Partial least squares regression score plot for combined analysis of stricture, proximal, and distal samples. F, Overlap of DEGs in stricture versus proximal margin, stricture versus distal margin, and proximal versus distal margin. G, Up and down regulation of 81 DEGs between stricture versus proximal margin and stricture versus distal margin.

The bulk transcriptomic analysis initially focused on comparing gene expression profiles between ileal stricture, non-strictured proximal margin, and non-strictured distal margin in pairwise fashion. Three hundred and three differentially expressed genes (DEGs) were found in the stricture versus proximal margin, 224 DEGs in the stricture versus distal margin, and 91 DEGs in the proximal versus distal margins (Supplementary File 1). As illustrated in the PCA score plots (Figure 1A–C), the gene expression profile of each site exhibited spatial separation from one to another, with significant differences in gene expression at each site. Similar results were found with supervised PLS analysis (Figure S1). However, upon analyzing all 3 regions collectively, the strictured samples formed a distinct cluster separate from proximal and distal samples, which by comparison overlapped in both unsupervised PCA modeling (Figure 1D) and supervised PLS-DA modeling (Figure 1E). To identify the genetic expression changes in stricture that were driving its separation from proximal and distal margins, we studied how the DEGs from the three pairwise comparisons overlapped. Eighty-one genes overlapped between stricture versus proximal margin and stricture versus distal margin (Figure 1F). Of these 81 genes, 64 resulted in up-regulated and 17 down-regulated in the stricture compared to either proximal or distal margin (Figure 1G).

#### Pathway enrichment analysis of DEGs revealed dysregulated pathways within strictures and their associated genes

**Figure 2.**
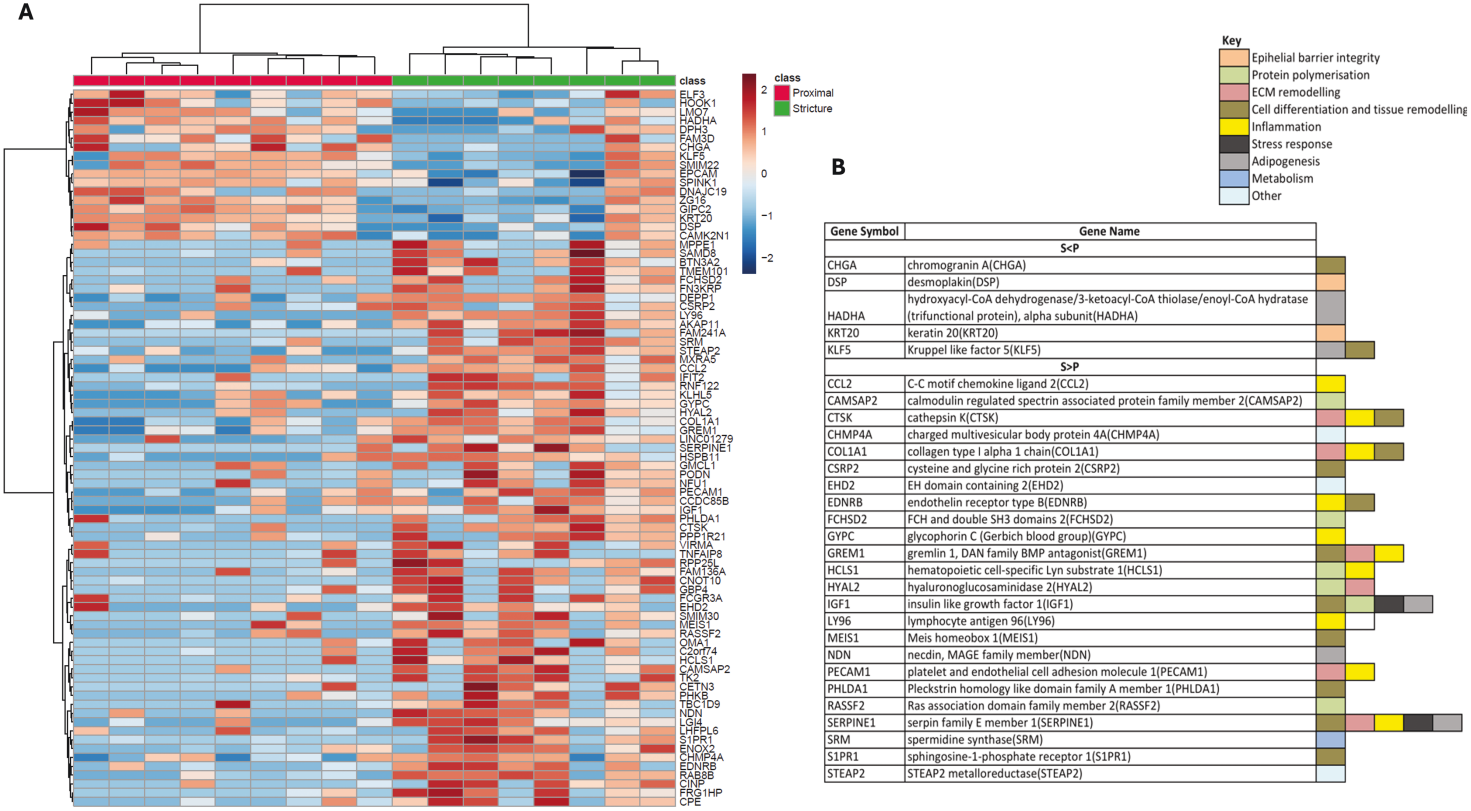
Pathway enrichment analysis of differentially expressed genes (DEGs). A, Heat-map showing differences of expression in ileal stricture compared to non-strictured proximal margin of the 81 previously identified DEGs in our CD population. B, Pathways enriched in the 81 DEGs and their associated genes.

Given the substantial overlap of gene expression between the proximal and distal margins compared to strictures, only the proximal margin was used as a comparator for pathway enrichment analysis. Thus, no cecal tissue was used in the comparison. The heat map in Figure 2A illustrates the consistent differences in expression of the 81 identified DEGs between strictures and the proximal margin across CD patients. Pathway enrichment analysis of the 81 DEGs revealed dysregulated pathways within strictures and their main associated genes, as depicted in Figure 2B and in Supplementary File 2. Among the enriched genes, 5 were down-regulated and 25 were up-regulated within strictures. Notably, 3 out of 5 down-regulated genes were linked to intestinal barrier integrity, including Krupple-like factor 5 (*KLF5*), the intermediate filament protein Keratin 20 (*KRT20*), and the desmosome factor desmoplakin (*DSK*). Conversely, the 25 up-regulated genes were primarily associated with pathways involving inflammation, protein polymerization, ECM remodeling, adipogenesis, and cellular stress. Upregulated genes related to ECM remodeling encompassed collagen type 1 alpha (*COL1A1*), its negative regulator cathepsin K (*CTSK*), hyaluronoglucosaminidase 2 (*HYAL2*), the bone morphogenic protein (BMP) antagonist Gremlin 1 (*GREM1*), and the serine protease inhibitor protein E1 (*SERPINE1*). Inflammation-associated genes included platelet and endothelial cell adhesion molecule 1 (*PECAM1*), the C-C motif chemokine ligand 2 (*CCL2*), the endothelin receptor type B (EDNRB), and lymphocyte antigen 96 (*LY96*). In addition, as concerns the adipogenesis pathway, the 2 genes insulin-like growth factor-1 (*IGF-1*) and necdin (*NDN*) were up-regulated, despite the hydroxyacyl-CoA dehydrogenase trifunctional multienzyme complex subunit alpha (HADHA) was down-regulated.

#### Association of LY96, AKAP11, SRM, GREM1, EHD2, SERPINE1, HDAC1, and FGF2 with strictures

**Figure 3.**
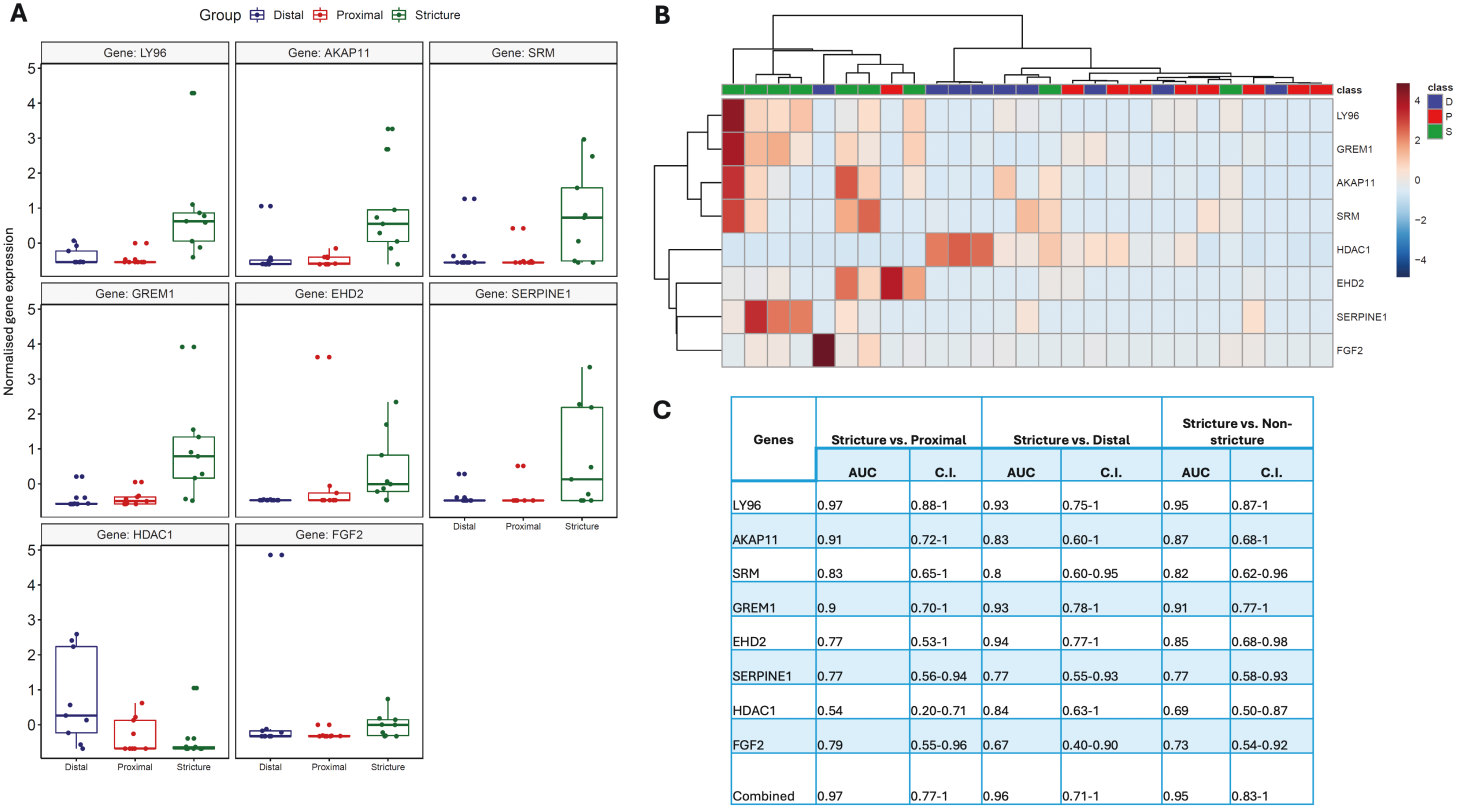
Genes associated with strictures. A, Box plots showing up- and down-regulated genes of normalized selected genes between stricture, non-strictured proximal margin, and non-strictured distal margin. B, Heatmap of selected gene expression across stricture [S], non-strictured proximal margin [P], and non-strictured distal margin [D]. C, Individual and combined AUC of selected genes from RNAseq analysis and their corresponding confidence interval. *P < .05; **P < .01; ns, not significant.

**Figure 4.**
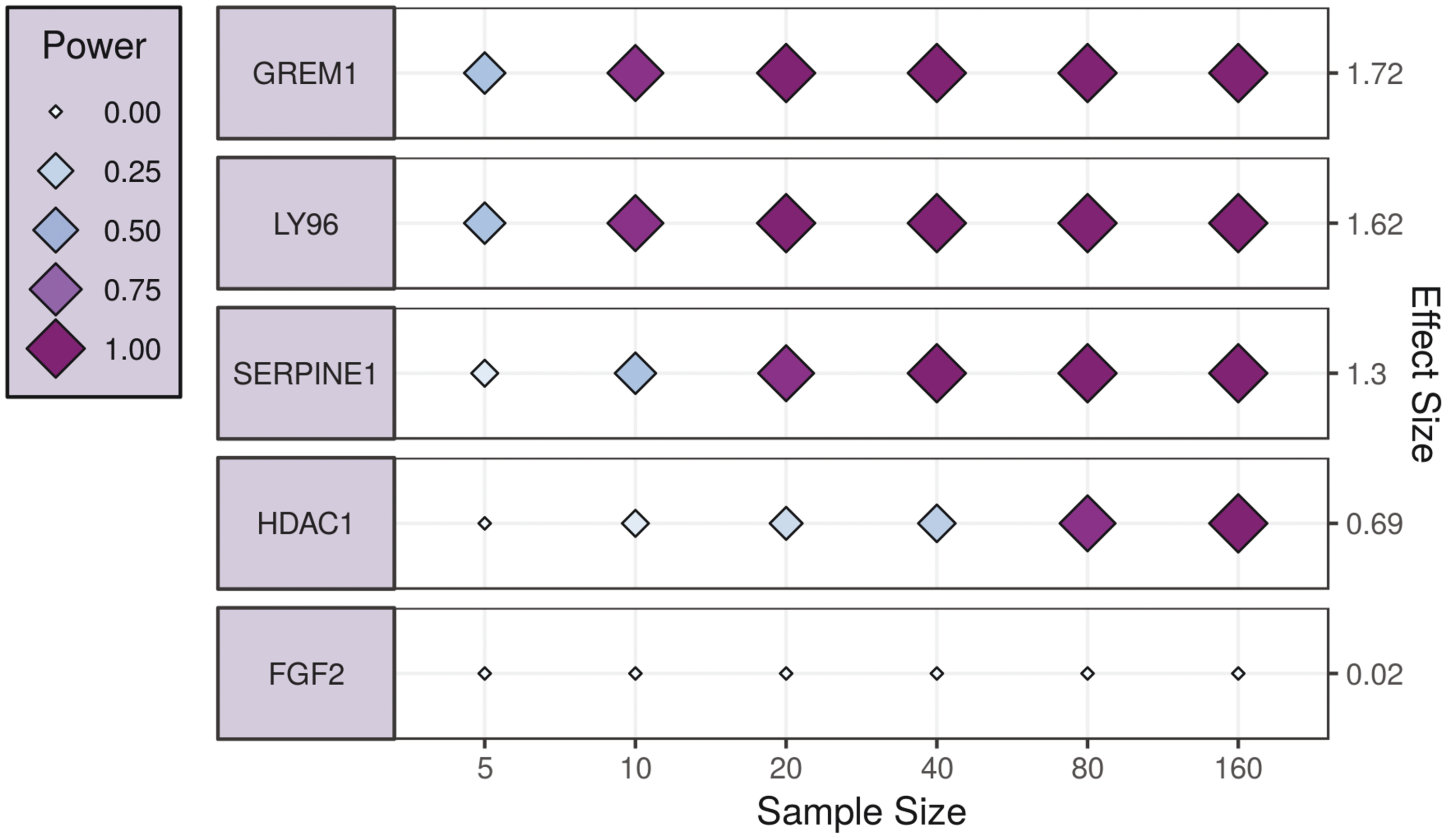
Estimated sample size and effect size of genes selected through bulk RNA sequencing. GREM1, LY96, and SERPINE1 exhibited a power of at least 75% to diagnose strictures within a sample size of 20 patients.

Starting from genes identified through PLS-DA analysis (Figure S1), those with a VIP score > 1 (Supplementary File 3) and deemed biologically relevant based on pathway enrichment analysis were selected to assess their specificity for strictures. These genes included *LY96*, AKAP11 (A-Kinase Anchoring Protein 11), *SRM* (Spermidine Synthase), *GREM1*, *EHD2* (EH Domain Containing 2), *SERPINE1*, *HDAC1* (Histone Deacetylase 1), and *FGF2* (Fibroblast Growth Factor 2). As depicted in Figure 3A and B, these 8 genes were differentially expressed in stricture compared to non-strictured margins. More in-depth, *LY96* (*P* < .01), *FGF2* (*P* < .01), *AKAP11* (*P* < .05), *GREM1* (*P* < .05), *EHD2* (*P* < .05), and *SERPINE1* (*P* < .01) were significantly up-regulated in strictures compared to both proximal and distal margin. Each gene achieved an AUC exceeding 0.69 in differentiating stricture, with a combined AUC of 0.95 (95% CI: 0.83-1) (Figure 3C). Furthermore, we estimated the required sample size to capture the effect size of these genes. Notably, *GREM1*, *LY96*, and *SERPINE1* exhibited a power of at least 75% to diagnose strictures within a sample size of 20 patients (Figure 4).

### qPCR Validation of DEGs Associated with Strictures from Bulk RNA Sequencing Analysis

**Figure 5.**
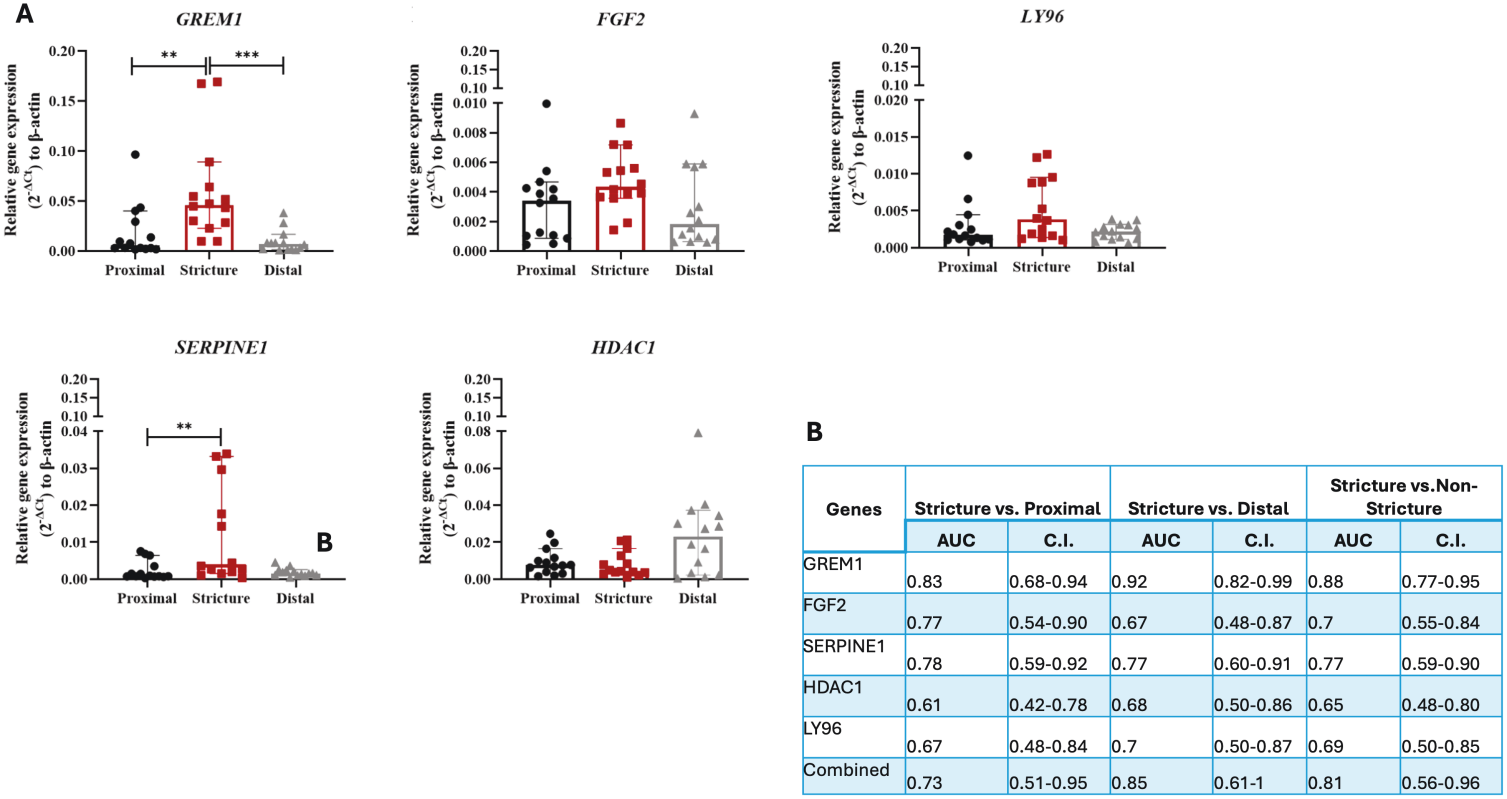
RT-qPCR analysis of genes differentiating stricture. A, Histograms showing up- and down-regulated genes of normalized selected genes between stricture, non-strictured proximal margin, and non-strictured distal margin. B, Individual and combined area under the curve (AUC) of selected genes from qPCR analysis and their corresponding confidence interval. **P < .01

For quantitative validation of selected DEGs identified from bulk RNAseq, RT-qPCR was conducted using samples from the nine patients enrolled for RNAseq and an additional 5 fibrostenotic CD surgical patients (Table S2). As illustrated in Figure 5, *GREM1* was confirmed significantly upregulated in strictures compared to both proximal and distal non-strictured margins (*P* < .01). Similarly, *SERPINE1* confirmed significant upregulation in strictures compared to the non-strictured proximal margin (*P* < .01). Although *LY96* and *FGF2* were confirmed upregulated in strictures, statistical significance was not reached. Furthermore, *HDAC1* was confirmed upregulated in distal and proximal non-strictured margins compared to strictures, without reaching statistical significance. Each gene achieved an AUC exceeding 0.65 in diagnosing stricture, with *GREM1* demonstrating the highest AUC (0.88 [0.77-0.95]). The combined AUC of 0.81 (95% CI: 0.56-0.96) confirmed the ability of these DEGs for identifying strictures (Figure 3C).

### LY96, GREM1, and Gene Combination Showed Promising Correlation with Strictures in a Publicly Available RNA-seq Dataset

When evaluating the association of the selected genes with stricture in a public dataset of CD-associated intestinal fibrosis, *LY96* and *GREM1* demonstrated reasonable ability to distinguish CD strictures. *LY96* achieved an AUC of 0.727 (95% CI: 0.56-0.89), while *GREM1* showed an AUC of 0.65 (95% CI: 0.47-0.81). When all genes were combined, the diagnostic performance resulted in an AUC of 0.620 (95% CI: 0.34-0.87) (Figure S2).

### Single-cell RNA Sequencing to Characterize Genes Specific for Strictures at Cellular Level

**Figure 6.**
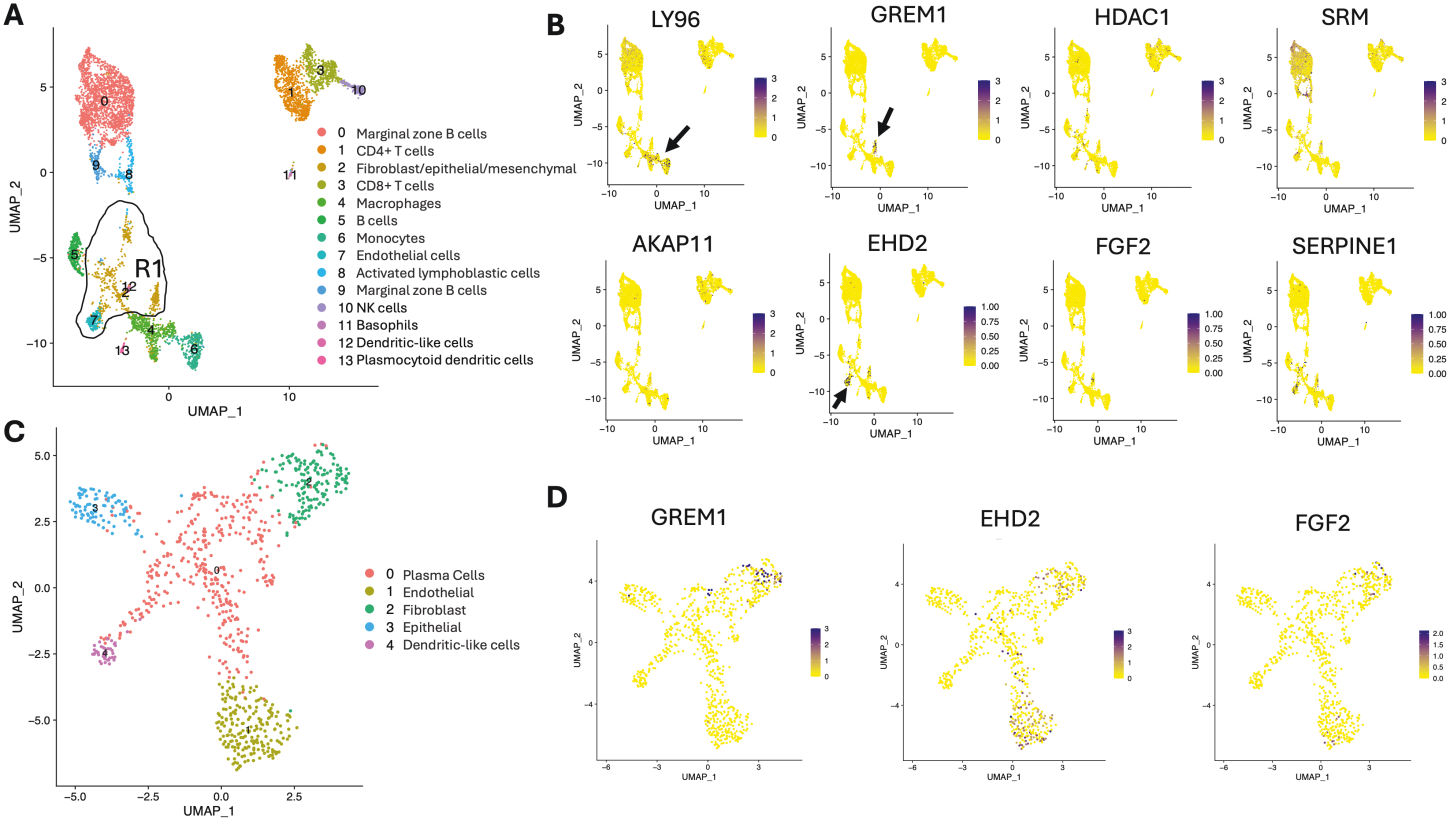
Single cell RNA sequencing of fibrostenotic CD. 10× scRNA-seq was performed on total cell populations (31.195 cells) isolated from stricture and non-strictured proximal margin of resected bowel of 3 CD patients. Single cells from all patients and locations were pooled and clustered using the UMAP_1 versus UMAP_2 parameters. A, Clusters were identified based on their expression of lineage-defining marker genes. B, UMAP_1 versus UMAP_2 expression plots for genes discriminating strictures and identified through bulk-RNAseq. R1 region showed clusters closely apposed in the UMAP_1 versus UMAP2 plot and containing cells with markers resembling fibroblasts, epithelial cells, endothelial cells, and dendritic cells. Arrows show cell clusters where the target gene is mainly expressed. C, Sub-clustering of R1 region. D, UMAP_1 versus UMAP_2 expression plots for genes associated with strictures, identified through bulk-RNAseq and expressed within R1 sub-clustered map.

To understand the role of the selected genes in stricture formation and delineate cell-specific changes and pathways contributing to the fibrotic phenotype, we conducted scRNA-seq on total single-cell populations isolated from stricture and non-strictured proximal margin samples in 3 CD patients from our cohort representing distal ileal fibrostenotic strictures (Table S3). Across all patients, we sequenced 31.195 cells, yielding 13 transcriptionally distinct clusters (Figure 6A), which we classified based on their expression of key lineage target genes (Figure S3). These clusters encompassed major immune cell subsets, including CD4+ and CD8+ T cells, natural killer cells, unswitched CD79A+CD20+ B cells —both CD27-naive cells and CD27+ memory cells—along with a significant population of switched antibody-secreting CD79AlowCD20-CD45—plasma cells. In addition, we identified myeloid populations such as CD14+ monocytes and closely related CD68+ macrophages, alongside two smaller populations resembling dendritic cells. Moreover, PECAM1(CD31) + CD34+ endothelial cells and a cluster (cluster 2) consisting of a mixture of EpCAM+ Vimlow epithelial cells and Thy1(CD90) + fibroblasts expressing collagen transcripts (COL1A1 and COL1A2) and platelet-derived growth factor receptor (PDGFR) were observed.

Following cluster definition, we analyzed the expression profiles of genes associated with strictures previously identified through bulk RNAseq (Figure 6B). *HDAC1*, *AKAP11*, and *SERPINE1* showed low expression across all cell subsets. *LY96*, consistent with its role in toll-like receptor (TLR) signaling, demonstrated upregulation in all professional antigen-presenting cells, including monocytes, macrophages, dendritic cells, memory B cells, and plasma cells. No significant difference in *LY96* expression was found between cells from strictures and those from non-strictured proximal margins (Figure S4A). Similarly, *SRM* showed expression in plasma cells, with no significant difference in expression between stricture cells compared to non-strictured proximal margin cells (Figure S4A). *GREM1*, *EHD2*, and FGF2 were mainly expressed in non-immune cells. To differentiate between cell types in cluster 2 and neighboring clusters 7 and 12 (region R1), we performed further sub clustering resulting in 5 populations (Figure 6C), that is, plasma cells, endothelial cells, fibroblasts, epithelial cells, and dendritic-like cells, based on lineage marker gene expression (Figure S5). Following sub clustering, we found *EHD2* and *FGF2* expression predominantly in fibroblasts and endothelial cells. Although not significant, *EHD2* exhibited higher expression in endothelial cells from strictures compared to those from non-strictured proximal margins, while no significant difference in *FGF2* expression was observed (Figure S4B). Notably, *GREM1* was exclusively expressed in fibroblasts (Figure 6D), with a trend to higher expression, albeit not significant, in fibroblasts from strictures compared to those from non-strictured segments (Figure S4B).

## Discussion

The multi-omics techniques can offer a comprehensive and in-depth characterization of intestinal tissue, holding immense potential to unravel underlying mechanisms of intestinal fibrosis and uncover novel therapeutic targets. Our study conducted a thorough transcriptomic analysis on a well-characterized relatively homogenous cohort of patients with CD undergoing surgery for ileal intestinal strictures with fresh samples collected from operating theater without delay. A novelty of the study lies in its specific focus on human tissue from fibrostenotic distal ileal CD, as well as the proximal and distal margins. The analysis exclusively targeted patients with end-stage fibrotic strictures devoid of inflammation. The bulk RNA transcriptomic analysis unveiled significant differences in gene expression and biologic pathways between fibrotic and non-fibrotic tissue, leading to the identification of key genes, that is, *LY96, AKAP11, SRM, GREM1, EHD2, SERPINE1, HDAC1,* and *FGF2*, which were suggested to be specific for strictures. This association was further validated through RT-qPCR. Moreover, leveraging scRNA-seq allowed us to elucidate distinct gene expression patterns at a cellular level, identifying potential biomarkers and therapeutic targets for CD-associated fibrosis.

Initially, the bulk RNA transcriptomic analysis identified 81 DEGs, 64 up-regulated, and 17 down-regulated in strictures underscoring the cellular reprogramming driving the fibrotic cascade and stenosis.

Notably, we observed the down-regulation of genes associated with intestinal barrier integrity, such as *KLF5*, known to stimulate the proliferation and migration of the intestinal epithelium,^14^ and *KRT20* and *DSK*, crucial components of epithelial adherens junctions vital for barrier integrity.^15^ These findings support the hypothesis of a role of barrier impairment in CD-related fibrosis, where the intestinal epithelial cells can release cytokines and growth factors promoting ECM deposition and can acquire a mesenchymal phenotype, directly contributing to fibrosis.^16^ In addition, emerging evidence suggests that necroptosis of epithelial cells following inflammatory injury may drive fibrosis.^17^ Consequently, restoring the intestinal epithelial barrier emerges as a promising approach in intestinal fibrosis. However, our study did not establish a causal relationship between barrier impairment and fibrosis development. Further research is therefore essential to clarify this connection and to deepen our understanding of the mechanisms involved.

Simultaneously, we noted the up-regulation of several genes in fibrotic strictures, related to ECM remodeling, inflammation, and adipogenesis. The up-regulation of genes associated with ECM remodeling and protein polymerization, such as *COL1A1*, its negative regulator *CTSK*, as well as *HYAL2*, which can degrade glycosaminoglycans, along with *GREM1* and *SERPINE1*, which inhibit ECM degradation, was anticipated in intestinal strictures. These genes may contribute to the imbalance between the production and degradation of collagen, a hallmark of intestinal fibrosis.^8^

Particularly intriguing was the finding of the up-regulated genes related to inflammation, including those associated with immune cell recruitment and migration,^18^ such as *PECAM1*, *CCL2* and *EDNRB*, as well as the gene *LY96* regulating TLR signaling and responsiveness to bacterial lipopolysaccharides.^19^ Despite careful clinical recruitment to include fibrotic non-inflammatory strictures and the histological confirmation of absence of overt inflammation based on appropriate scoring, an inflammatory genetic signature was still evident in our strictures. This observation highlights the presence of chronic subtle inflammation in long-standing fibrostenotic disease, as suggested by our recent studies.^6^

Also noteworthy was the dysregulation of the genes associated with adipogenesis pathway. Within the fibrotic strictures, the up-regulation of *IGF-1*, which regulates brown adipose tissue (BAT) and promotes adipogenesis in white adipose tissue (WAT),^20^ was observed. The *IGF-1* effect is mediated by the adipogenic suppressor *NDN*,^21^ up-regulated in intestinal strictures. Furthermore, the gene encoding the trifunctional protein HADHA, catalyzing the last steps of beta oxidation of long-chain fatty acids, resulted down-regulated in strictures.^22^ Collectively, these genetic changes suggest altered adipogenesis in strictures, characterized by reduced BAT and enhanced WAT adiposity. Such dysregulation of the adipogenesis pathway may contribute to developing creeping fat, an increasingly studied therapeutic target in intestinal fibrosis.^23^

It is worth noting that some expected genes—such as those associated with TGF-β, α-smooth muscle actin, matrix metalloproteinases, and their inhibitors—along with pathways commonly linked to fibrosis, did not show significant differences between strictured and non-strictured segments. This underscores potential limitations of the dataset, primarily attributable to the limited sample size. In addition, the end-stage of fibrosis in the samples may have influenced the activation or suppression of specific pathways.

Currently, there is a notable absence of biomarkers for identifying stricturing Crohn’s disease in clinical practice and clinical trials,^24,25^ which underlines a significant unmet need in the field. Previous studies have focused on individual genes, such as *NOD2*, for fibrosis diagnosis,^26^ lacking a comprehensive genomic evaluation of fibrotic disease as undertaken in this study. Utilizing a machine-learning model and pathway enrichment analysis to consider the biological relevance of genes, we identified targeted genes capable of accurately discriminating strictures. *LY96, AKAP11, SRM, GREM1, EHD2, SERPINE1, HDAC1,* and *FGF2* demonstrated considerable association with the stricturing phenotype, with robust individual and combined AUC values. These findings were further supported by qPCR validation. Pending further validation of our results, the integration of machine learning-guided genomics holds promise for translation into clinical trials and clinical practice, aiding in patient stratification.

We used a publicly available dataset to further validate the correlation of the targeted genes with strictures. *LY96*, *GREM1*, and the combination of all targeted genes demonstrated a reasonable discriminative performance. Nonetheless, it is important to note that no existing studies to date replicate the specific design of our study and the specific population recruited, which carefully selected fibrotic patients without ongoing inflammation and used surgical specimens from fibrotic strictures along with their proximal and distal non-fibrotic margins. These differences may have influenced the results of this analysis, warranting future prospective studies for further validation.

Our study was bolstered by the in-depth characterization of targeted gene expression profiles across distinct cell types through scRNA-seq. Significantly, *GREM1*, the most potent gene associated with stricture development in our machine learning analysis, was exclusively up regulated in stricture fibroblasts. GREM1 is an antagonist of the BMP family members, contributing to tissue development and differentiation.^27^ Previous research has elucidated GREM1’s role as a ligand for the vascular endothelial growth factor receptor 2 (VEGFR2), triggering downstream MAPK signaling.^28^ Notably, GREM1 up-regulation in intestinal fibrotic tissue has been observed in murine models, with functional assays demonstrating its capacity to stimulate the proliferation and activation of intestinal fibroblasts through enhanced fatty acid oxidation.^29^ GREM1+ fibroblasts have emerged as an intriguing therapeutic target in intestinal fibrosis, with different avenues available for specific targeting, including GREM1-neutralizing antibodies and VEGFR2-specific inhibitors.^29,30^ It is worth noting that, differently from bulk sequencing, scRNA-seq did not reveal a significant difference in GREM-1 expression between fibroblasts from the stricture and its margin. We hypothesize that this discrepancy may arise from differences in the number of cells expressing GREM-1 rather than variations in per-cell expression levels between the stricture and its margin. In addition, the small sample size may have impacted our findings.

Two additional genes, *FGF2* and *EHD2*, exhibited predominant upregulation in fibroblasts and endothelial cells in scRNA-seq analysis. *FGF2* is crucial in various biological processes, including tissue repair, fibroblast proliferation, and angiogenesis.^31^ Preliminary investigations have reported elevated serum levels of FGF2 in CD patients with intestinal strictures, suggesting its potential involvement in fibrogenesis.^32^ Our study provides cellular-level evidence implicating FGF2 in CD intestinal fibrosis, likely regulating fibroblast activation, extracellular matrix (ECM) production, and endothelial-to-mesenchymal transition. Hence, targeting FGF2 holds promise, as demonstrated in cardiac fibrosis studies.^33^ As for EHD2, previous studies have linked this gene, which encodes a membrane transport regulatory protein, with mouse embryonic fibroblasts and liver cirrhosis.^34^ Our study is the first to establish a correlation between EHD2 and intestinal fibrosis in humans, prompting further exploration of its potential as a biomarker and therapeutic target.

In our scRNA-seq analysis, *LY96*, known for its involvement in inflammation through TLR signaling,^35^ and SRM, which encodes the natural polyamine spermidine primarily implicated in autophagy and is associated with TGF-β1 signaling,^36^ were found to be up-regulated in immune cells within intestinal strictures. This finding underscores the intricate composition of strictured segments and the significant interaction between stromal and immune cells,^4^ with IL-34 playing a pivotal role in mediating their cross-talk.^37^

*HDAC1, AKAP11,* and *SERPINE1* exhibited less cell-specific expression compared to other genes related to strictures, with low expression observed across all cell subsets. *HDAC1* encodes an epigenetic master regulator recently linked to stricturing Crohn’s disease, suggesting potential for *HDAC1* inhibitor therapy to reverse fibrosis-associated epigenetic changes.^38^ While *AKAP11*, encoding a structural protein binding the regulatory subunit of protein kinase A, has not been directly associated with fibrosis, targeting another protein in the same family, AKAP12, has shown promise in liver fibrosis inhibition,^39^ indicating potential for *AKAP11* in intestinal fibrosis. *SERPINE1*, encoding plasminogen activator inhibitor-1 (PAI-1), has been previously observed to be up-regulated in inflamed Crohn’s disease mucosa.^40^ Increased PAI-1 transcripts have been detected in active fibrotic bowel lesions, and inhibition through TM5275 has demonstrated efficacy in attenuating fibrogenesis in mouse models,^41^ suggesting promise as an anti-fibrotic drug.

It is important to acknowledge that bulk transcriptomics and scRNA-seq are fundamentally different techniques, making direct comparisons challenging. Notably, the tissue processing required for scRNA-seq can result in reduced cell viability and the loss of up to 60% of tissue cells during the digestion of tissue. This may have limited our analysis and could explain why genes that are highly differentially expressed in bulk sequencing appear to have low expression in scRNA-seq.

The scRNA-seq was conducted as an exploratory analysis and requires future functional validation in larger prospective studies. Recent scRNA-seq studies, despite being based on a small sample size and using a different study design, have identified specific molecular fingerprints of fibrotic strictures and uncovered the heterogeneity of fibroblasts within intestinal strictures.^10^ Interestingly, *GREM1*+ fibroblasts have emerged in other studies as key regulators of intestinal fibrosis, which supports our findings and strengthens the case for *GREM1* as a promising therapeutic target.^11,42^

Our study has certain limitations. Firstly, it is constrained by a small sample size, resulting from the premature closure of the study due to the COVID-19 pandemic. Nevertheless, the study’s strength lies in meticulously recruiting CD patients, specifically selecting patients with a confirmed stricturing phenotype and excluding patients with inflammatory phenotype at MRE and histology, supported by inflammatory markers. Another notable strength is the comprehensive genomic assessment of fibrostenotic CD, incorporating bulk transcriptomic analysis, qPCR validation, and deeper evaluation through scRNA-seq. While scRNA-seq was conducted on a limited number of patients, the phenotype was uniformly fibrostenotic. Despite these limitations, we could analyze a sufficient number of cells with homogenous distribution, allowing a robust analysis. It is important to note that this study solely provides a genomic-wide characterization of fibrosis in CD, identifying genes associated with intestinal strictures. Another important limitation involves the selection of non-strictured margins, as distal margins could contain colonic rather than ileal tissue, and some proximal margins may have been dilated due to the stricture. While these factors could have influenced the transcriptomic profiles at these sites, our PCA and PLS-DA analyses revealed overlapping DEGs between proximal and distal margins, along with uniform expression across all patients, suggesting a consistent genetic profile within our population.

A key strength of our study lies in the in-depth genetic characterization using scRNA-seq combined with machine learning models to identify potential therapeutic targets. scRNA-seq has proven highly effective in mapping tissue architecture, identifying critical cells involved in fibrosis, detailing key cellular interactions, and revealing the molecular pathways at play.^43^ This approach enhances our understanding of intestinal fibrosis and helps uncovering novel therapeutic targets. However, the translation of scRNA-seq from bench to bedside is still limited by its high costs, the need for standardization, and the specialized expertise required. In addition, it generates vast amounts of data that are difficult for humans to interpret. In this context, machine learning models offer valuable support by standardizing and analyzing OMICs and multi-OMICs data, providing critical insights for researchers and clinicians. Indeed, the application of artificial intelligence models to OMIC data in fibrosis has already shown promise in identifying key cellular and molecular subsets involved in the fibrotic process,^44,45^ potentially paving the way for personalized medicine in this field.

While our results represent an intriguing advance in the study of intestinal fibrosis in IBD, further prospective studies with larger sample sizes are needed for further validation and to enhance the generalizability of the findings. Also, future longitudinal studies are warranted to explore the dynamic changes in gene expression associated with disease progression over time. Future experimental validation is necessary to confirm the roles of individual genes and assess their potential as novel therapeutic targets. This includes proteomic analyses to measure gene expression and functional studies to assess the impact of gene suppression for therapeutic purposes.

## Conclusion

Our study provides a comprehensive transcriptomic characterization of fibrostenotic CD, revealing novel differences in biological pathways and gene expression between strictures and non-strictured tissue in CD patients. Using a machine-learning model, we have identified targeted genes that can accurately distinguish intestinal strictures alone and combined, offering promising biomarkers and potential therapeutic targets in fibrostenotic CD. This research represents another step in unraveling the intricate puzzle of intestinal fibrosis, enhancing our understanding of underlying mechanisms and contributing to the enhancement of clinical trials and clinical practice.

## Supplementary data

Supplementary data is available at *Inflammatory Bowel Diseases* online.

izaf021_suppl_Supplementary_Material

## Data Availability

The scRNA-seq and bulk transcriptomic data are publicly available online at https://doi.org/10.6084/m9.figshare.28067543.v2. For any additional inquiries, please contact the corresponding author.
