# Supplementary material for: Novel Transcriptomic Signatures in Fibrostenotic Crohn’s Disease: Dysregulated Pathways, Promising Biomarkers, and Putative Therapeutic Targets
Source: Inflamm Bowel Dis. 2025 Feb 20;31(6):1502–13. doi: 10.1093/ibd/izaf021 (PMC12166298; doi:10.1093/ibd/izaf021)
Supplement: izaf021_suppl_Supplementary_Material [file izaf021_suppl_supplementary_material.zip › IBDJNL_izaf021_suppl_Figures1-5, Files 1-3, Tables 1-3, Captions/Supplementary Legends.docx]

**Supplementary Figure captions**

**Supplementary Figure 1. Partial least square score plots for DEGs.** (A-C) Supervised partial least square score (PLS) score plots performed on the differentially expressed transcriptome datasets demonstrating clustering and variation of subjects within cohorts, i.e. i.e. stricture vs proximal margin (A), stricture vs distal margin (B) and proximal vs distal margin (C). The dots represent samples and are colored according to the subject cohort. Ellipses represent 95% confidence. Results are plotted according to the PC1 and PC2 scores, with the percent variation explained by the respective axis. (D) PLS model quality explained by the model variance (R^2^) and predictive ability (Q^2^) of each comparison.

**Supplementary Figure 2. Genes associated with strictures in a public RNA-seq dataset.** (A) Heatmap of selected gene expression across fibrotic and not fibrotic samples from a public bulk RNA-seq dataset (GSE192786). (B) Individual and combined area under the curve (AUC) of selected genes from RNAseq analysis and their corresponding confidence interval (CI).

**Supplementary Figure 3. Clusters of cells identified through single cell RNA sequencing analysis from specimens of patients with fibrostenotic Crohn’s disease.** 10X scRNA-seq was performed on total cell populations (31.195 cells) isolated from stricture and non-strictured proximal margin of resected bowel of 3 CD patients. Single cells from all patients and locations were pooled and clustered using the UMAP_1 vs UMAP_2 parameters. (A) UMAP plots showing different clusters of cells identified according to their expression of key lineage target genes. (B) List of key lineage target genes used for cluster classification.

**Supplementary Figure 4. UMAP plots and violin plot showing different expression of targeted genes between cells from strictures vs cells from non-strictured proximal margin.** (A) Clusters identified in the whole cellular population based on their expression of lineage defining marker genes. On the right, UMAP and violin plots showing different expression of *LY96* and *SRM* between stricture and non-strictured immune cells. (B) UMAP plot showing sub-clustering of R1 region. On the right, UMAP plots and violin plots showing different expression of *GREM1* between stricture and non-strictured fibroblasts and different expression of EHD2 and FGF2 between stricture and non-strictured fibroblasts and endothelial cells.

**Supplementary Figure 5. Sub-clustering of cells identified through single cell RNA sequencing analysis in cluster 2, 7 and 12 and positioned close by (region R1) in the UMAP_1 vs UMAP_2 plot (Fig 5A).** (A) UMAP plot showing sub-clustering of R1 region. (B) List of key lineage target genes used for cluster classification. (C) UMAP plots showing different clusters of cells identified according to their expression of key lineage target genes.

**Supplementary File captions**

**Supplementary file 1.** Differentially expressed genes between ileal stricture, non-strictured proximal margin and non-strictured distal margin.

**Supplementary file 2.** Genes associated with the most enriched pathways in the 81 differentially expressed genes between stricture and non-strictured margins.

**Supplementary fie 3.** Genes expression based on VIP scores.
