# Supplementary material for: Novel Transcriptomic Signatures in Fibrostenotic Crohn’s Disease: Dysregulated Pathways, Promising Biomarkers, and Putative Therapeutic Targets
Source: Inflamm Bowel Dis. 2025 Feb 20;31(6):1502–13. doi: 10.1093/ibd/izaf021 (PMC12166298; doi:10.1093/ibd/izaf021)
Supplement: izaf021_suppl_Supplementary_Material [file izaf021_suppl_supplementary_material.zip › IBDJNL_izaf021_suppl_Figures1-5, Files 1-3, Tables 1-3, Captions/Supplementary Table 1_final.docx]

**Supplementary Table 1. Adapter Sequences used for scRNAseq**

| **Sample** | **Adapter ID** | **Adapter sequences** | | | |
| --- | --- | --- | --- | --- | --- |
| Patient1_proximal_sort-1 | SI-GA-B6 | CGTTAATC | GCCACGCT | TTACTCAG | AAGGGTGA |
| Patient1_proximal_sort-2 | SI-GA-H5 | CCACTACA | GATTCTGG | TGCGGCTT | ATGAAGAC |
| Patient1_stricture_sort-1 | SI-GA-G5 | GAGCAAGA | TCTGTGAT | CGCAGTTC | ATATCCCG |
| Patient1_ stricture _sort-2 | SI-GA-F5 | GACTACGT | CTAGCGAG | TCTATATC | AGGCGTCA |
| Patient2_proximal_sort-1 | SI-GA-C6 | ATTACTTC | TGCGAACT | GCATTCGG | CAGCGGAA |
| Patient2_proximal_sort-2 | SI-GA-D6 | CATGCGAT | TGATATTC | GTGATCGA | ACCCGACG |
| Patient2_ stricture _sort-1 | SI-GA-E6 | CTGCGGCT | TGATATTC | AGAAACTC | TCTGTTGG |
| Patient2_ stricture _sort-2 | SI-GA-F6 | CGGAGCAC | GACCTATT | ACTTAGGA | TTAGCTCG |
| Patient3_proximal | SI-GA-A1 | GGTTTACT | CTAAACGG | TCGGCGTC | AACCGTAA |
| Patient3_stricture | SI-GA-B1 | GTAATCTT | TCCGGAAG | AGTTCGGC | CAGCATCA |
