# Supplementary material for: Novel Transcriptomic Signatures in Fibrostenotic Crohn’s Disease: Dysregulated Pathways, Promising Biomarkers, and Putative Therapeutic Targets
Source: Inflamm Bowel Dis. 2025 Feb 20;31(6):1502–13. doi: 10.1093/ibd/izaf021 (PMC12166298; doi:10.1093/ibd/izaf021)
Supplement: izaf021_suppl_Supplementary_Material [file izaf021_suppl_supplementary_material.zip › IBDJNL_izaf021_suppl_Figures1-5, Files 1-3, Tables 1-3, Captions/Supplementary Table 2_final.docx]

**Supplementary Table 2. Patients considered for RT-qPCR**

| Fibrostenotic CD patients undergoing surgery, N=14 | |
| --- | --- |
| Clinical characteristics | |
| Age (years), median (IQR) | 35 (25-54) |
| F/M (ratio) | 5/9 |
| Disease duration (years) median (IQR) | 14 (6-20) |
| Montreal Classification |  |
| Age at diagnosis, n (%) |  |
| A1 | 4 (29) |
| A2 | 9 (64) |
| A3 | 0 |
| Location, n (%) |  |
| L1 | 8 (57) |
| L2 | 0 |
| L3 | 6 (43) |
| Behaviour, n (%) |  |
| B1 | 0 |
| B2 | 14 (100) |
| B3 | 0 |
| Perianal disease | 2 (14) |
| Previous bowel surgery for CD, n (%) | 4 (29) |
| Medication before surgery | |
| Mesalamine, n % | 1 (7) |
| Steroid, n % | 4 (29) |
| Immunosuppressive, n % | 3 (21) |
| Anti-TNF, n % | 5 (36) |
| Vedolizumab, n % | 0 |
| Ustekinumab, n % | 3 (21) |
| Bio-experienced, n % | 6 (43) |
| Lab results before surgery | |
| Hb g/L, median (IQR) | 137 [128-142] |
| CRP mcg/mL, median (IQR) | 5 [1.5-24.5] |
| Albumin g/dL, median (IQR) | 42 [39.5-44] |
| Faecal calprotectin, median (IQR) | 142 (75-249) |
| Type of surgery | |
| Ileo-caecal resection, n (%) | 4 (29) |
| Right hemicolectomy, n (%) | 3 (21) |
| Ileal resection, n (%) | 7 (50) |

*Abbreviations: CD, Crohn’s disease; CRP, C-reactive protein; F, female; Hb, haemoglobin; IQR, interquartile range; M, male; TNF, tumour necrosis factor.*
