# Supplementary material for: Novel Transcriptomic Signatures in Fibrostenotic Crohn’s Disease: Dysregulated Pathways, Promising Biomarkers, and Putative Therapeutic Targets
Source: Inflamm Bowel Dis. 2025 Feb 20;31(6):1502–13. doi: 10.1093/ibd/izaf021 (PMC12166298; doi:10.1093/ibd/izaf021)
Supplement: izaf021_suppl_Supplementary_Material [file izaf021_suppl_supplementary_material.zip › IBDJNL_izaf021_suppl_Figures1-5, Files 1-3, Tables 1-3, Captions/Supplementary Table 3_final.docx]

**Supplementary Table 3. Patients considered for scRNAseq**

| Patient | Age | Sex | Disease duration (y) | Montreal classification | Medications before surgery | Type of surgery |
| --- | --- | --- | --- | --- | --- | --- |
| 1 | 54 | M | 21 | A2L1B2 | None | Ileal resection |
| 2 | 55 | F | 36 | A2L1B2 | Anti-TNF | Ileo-caecal resection |
| 3 | 26 | M | 12 | A1L1B2 | Anti-TNF and Thiopurine | Right hemicolectomy |

*Abbreviations: A, age of onset; B, disease behavior; F, female; L, disease location; M, male; TNF, tumor necrosis factor.*
