# Supplementary figures and images for: Novel Transcriptomic Signatures in Fibrostenotic Crohn’s Disease: Dysregulated Pathways, Promising Biomarkers, and Putative Therapeutic Targets
Source: Inflamm Bowel Dis. 2025 Feb 20;31(6):1502–13. doi: 10.1093/ibd/izaf021 (PMC12166298; doi:10.1093/ibd/izaf021)

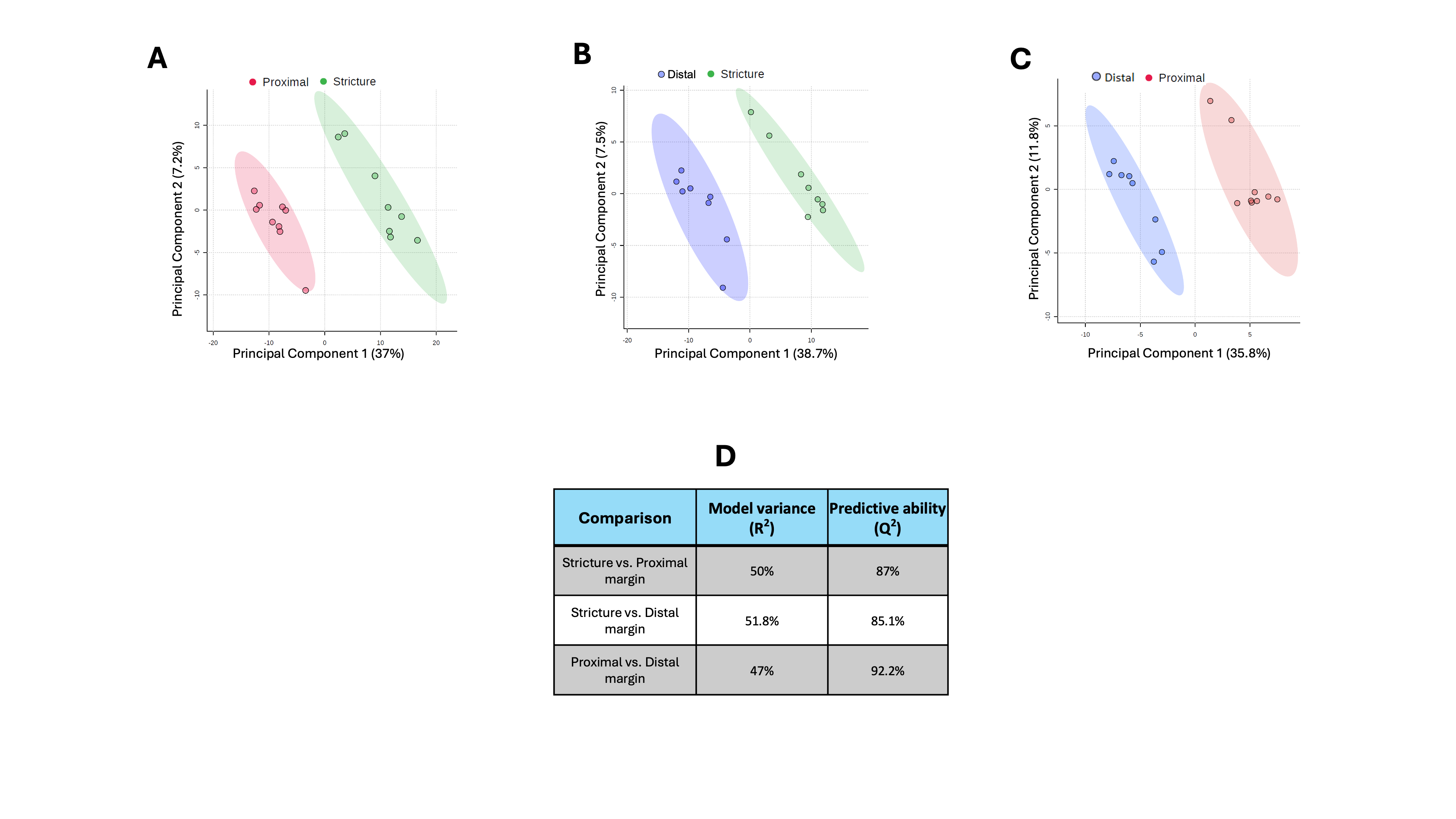

Supplement: izaf021_suppl_Supplementary_Material [file izaf021_suppl_supplementary_material.zip › IBDJNL_izaf021_suppl_Figures1-5, Files 1-3, Tables 1-3, Captions/Supp Fig 1_R2.tiff]

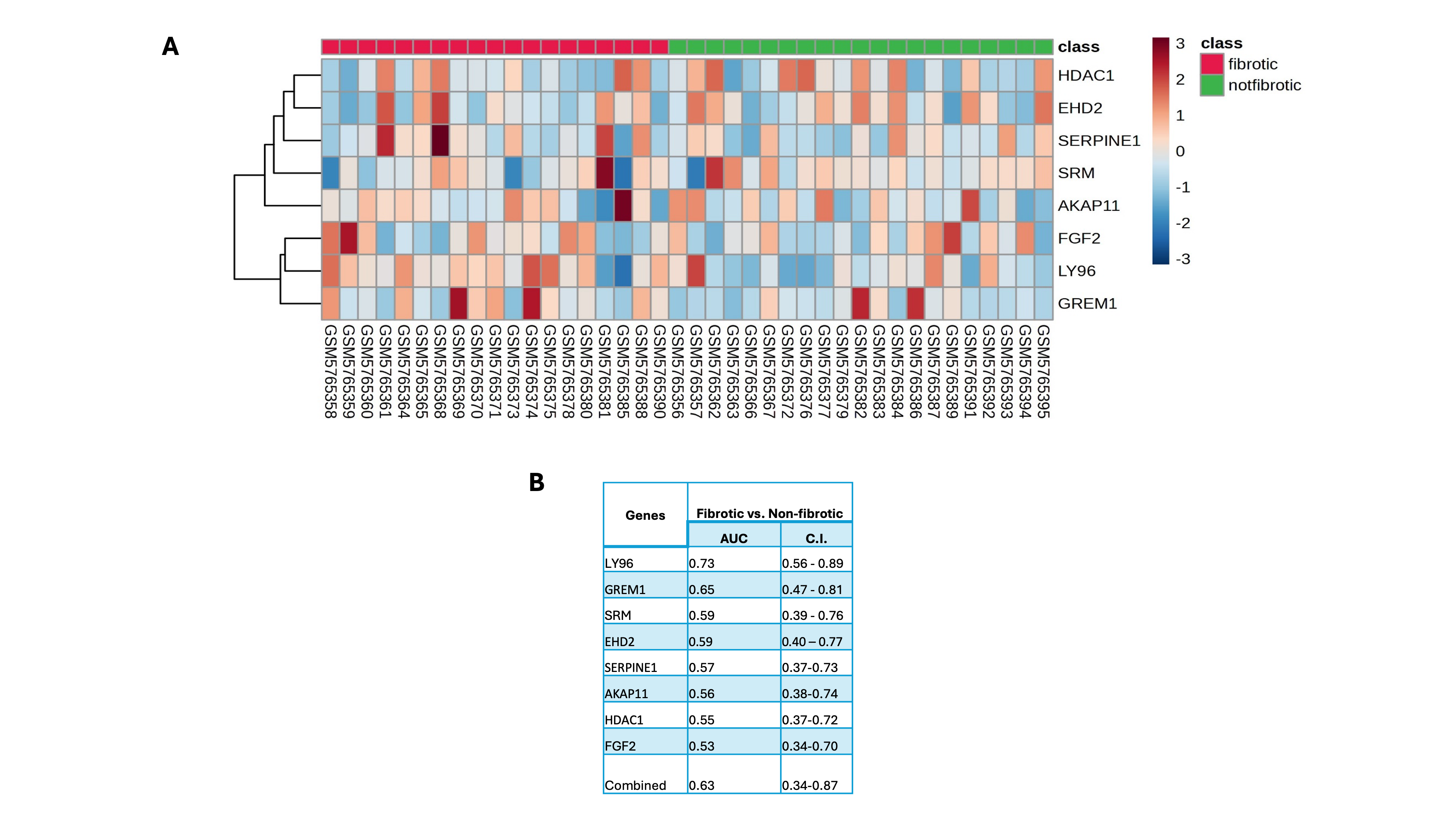

Supplement: izaf021_suppl_Supplementary_Material [file izaf021_suppl_supplementary_material.zip › IBDJNL_izaf021_suppl_Figures1-5, Files 1-3, Tables 1-3, Captions/Supp Fig 2_R2.tiff]

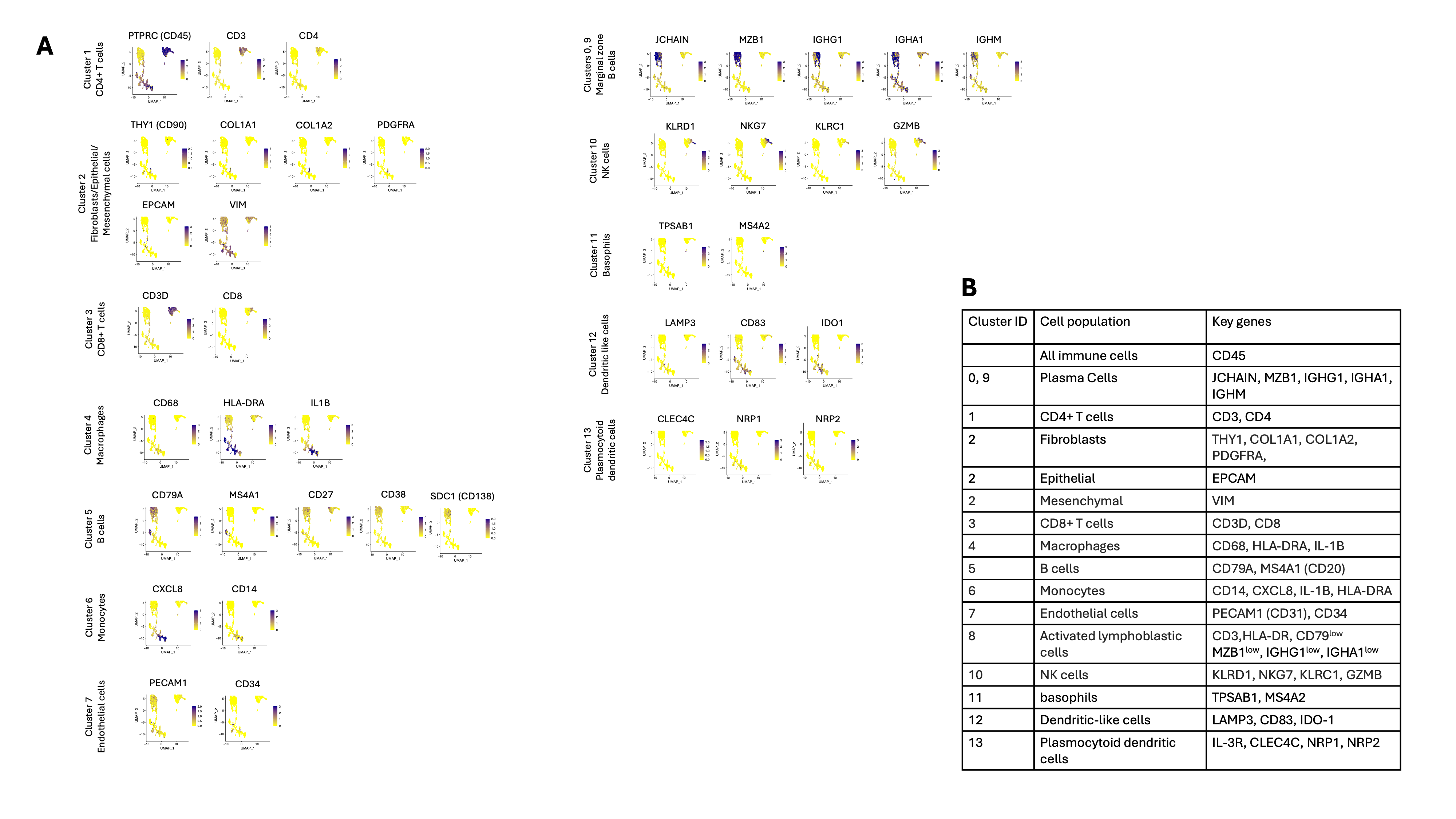

Supplement: izaf021_suppl_Supplementary_Material [file izaf021_suppl_supplementary_material.zip › IBDJNL_izaf021_suppl_Figures1-5, Files 1-3, Tables 1-3, Captions/Supp Fig 3_R2.tiff]

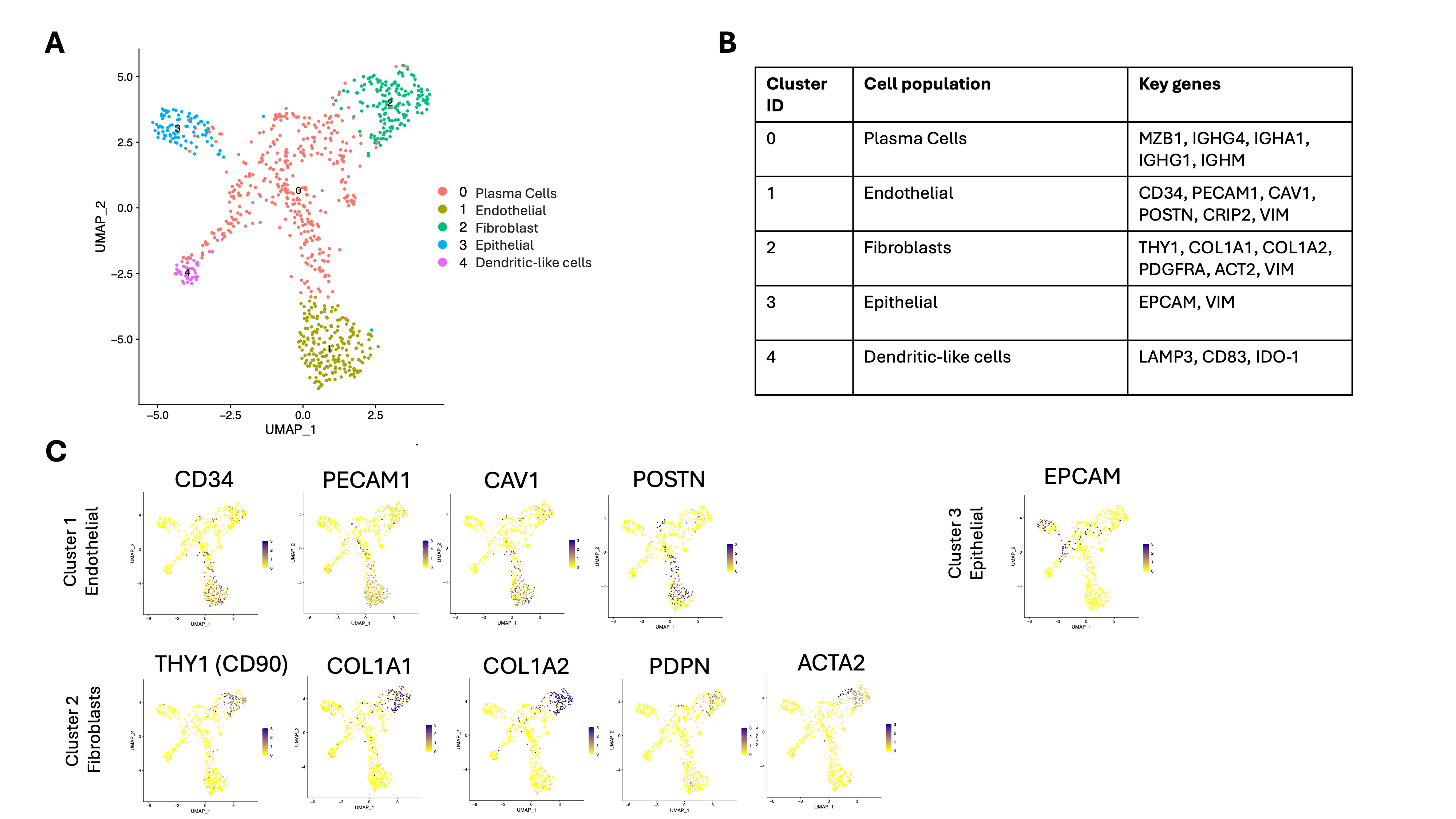

Supplement: izaf021_suppl_Supplementary_Material [file izaf021_suppl_supplementary_material.zip › IBDJNL_izaf021_suppl_Figures1-5, Files 1-3, Tables 1-3, Captions/Supp Fig 4_R2.tiff]

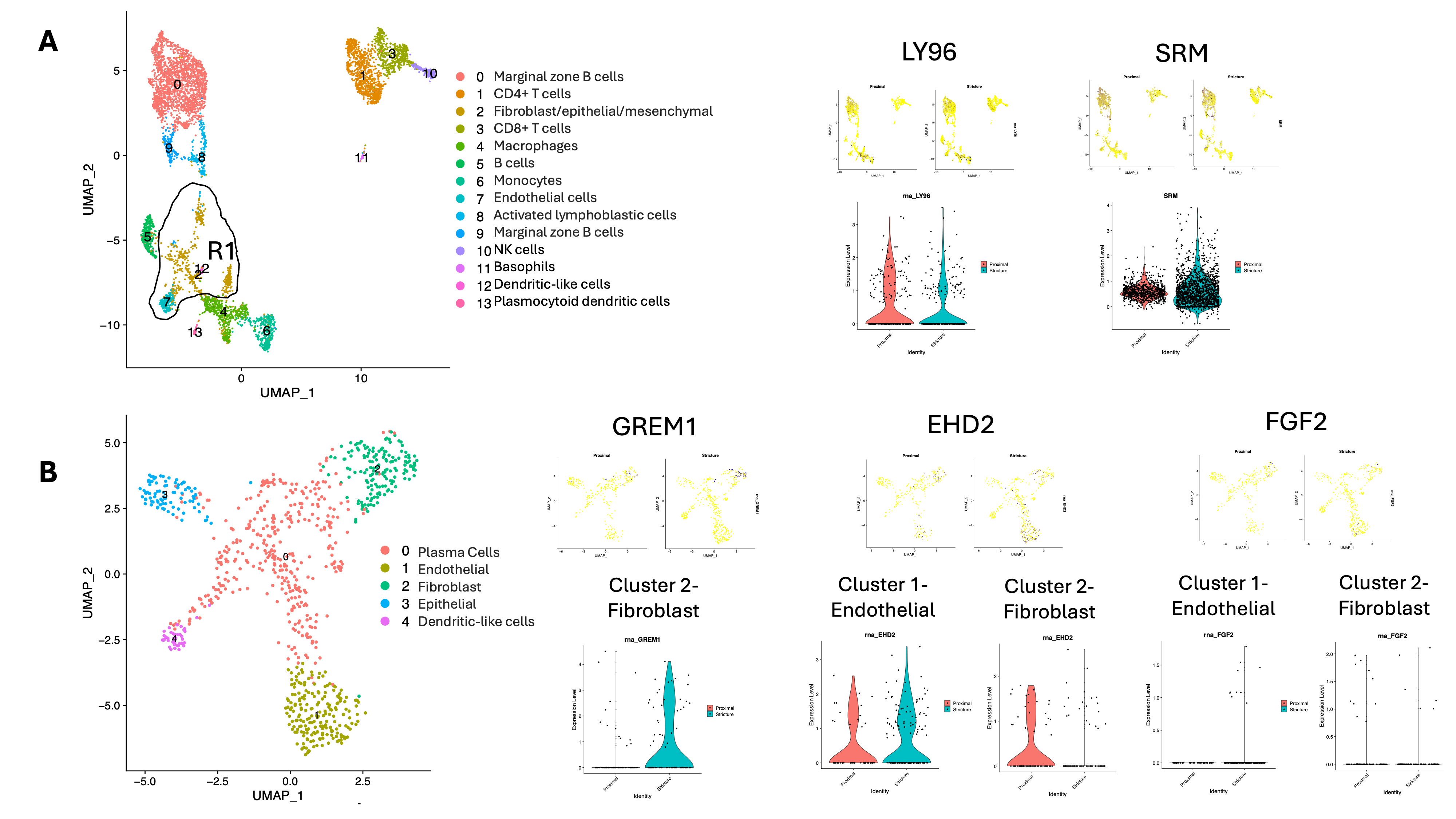

Supplement: izaf021_suppl_Supplementary_Material [file izaf021_suppl_supplementary_material.zip › IBDJNL_izaf021_suppl_Figures1-5, Files 1-3, Tables 1-3, Captions/Supp Fig 5_R2.tiff]
